# Identification of human-to-human transmissibility factors in PB2 proteins of influenza A by large-scale mutual information analysis

**DOI:** 10.1186/1471-2105-9-S1-S18

**Published:** 2008-02-13

**Authors:** Olivo Miotto, AT Heiny, Tin Wee Tan, J Thomas August, Vladimir Brusic

**Affiliations:** 1 Department of Biochemistry, Yong Loo Lin School of Medicine, National University of Singapore, 8 Medical Drive, Singapore; 2 Institute of Systems Science, National University of Singapore, 25 Heng Mui Keng Terrace, Singapore; 3 Department of Pharmacology and Molecular Sciences, Johns Hopkins University School of Medicine, 725 North Wolfe Street, Baltimore, USA; 4 Cancer Vaccine Center, Dana-Farber Cancer Institute, 77 Avenue Louis Pasteur, Boston, USA; 5 School of Land, Food, and Crop Sciences, University of Queensland, Brisbane 4072, Australia

## Abstract

**Background:**

The identification of mutations that confer unique properties to a pathogen, such as host range, is of fundamental importance in the fight against disease. This paper describes a novel method for identifying amino acid sites that distinguish specific sets of protein sequences, by comparative analysis of matched alignments. The use of mutual information to identify distinctive residues responsible for functional variants makes this approach highly suitable for analyzing large sets of sequences. To support mutual information analysis, we developed the AVANA software, which utilizes sequence annotations to select sets for comparison, according to user-specified criteria. The method presented was applied to an analysis of influenza A PB2 protein sequences, with the objective of identifying the components of adaptation to human-to-human transmission, and reconstructing the mutation history of these components.

**Results:**

We compared over 3,000 PB2 protein sequences of human-transmissible and avian isolates, to produce a catalogue of sites involved in adaptation to human-to-human transmission. This analysis identified 17 characteristic sites, five of which have been present in human-transmissible strains since the 1918 Spanish flu pandemic. Sixteen of these sites are located in functional domains, suggesting they may play functional roles in host-range specificity. The catalogue of characteristic sites was used to derive sequence signatures from historical isolates. These signatures, arranged in chronological order, reveal an evolutionary timeline for the adaptation of the PB2 protein to human hosts.

**Conclusion:**

By providing the most complete elucidation to date of the functional components participating in PB2 protein adaptation to humans, this study demonstrates that mutual information is a powerful tool for comparative characterization of sequence sets. In addition to confirming previously reported findings, several novel characteristic sites within PB2 are reported. Sequence signatures generated using the characteristic sites catalogue characterize concisely the adaptation characteristics of individual isolates. Evolutionary timelines derived from signatures of early human influenza isolates suggest that characteristic variants emerged rapidly, and remained remarkably stable through subsequent pandemics. In addition, the signatures of human-infecting H5N1 isolates suggest that this avian subtype has low pandemic potential at present, although it presents more human adaptation components than most avian subtypes.

## Background

In the study of pathogens, it is fundamentally important to identify the molecular elements that enable transmission and replication in humans, and understand their evolutionary patterns as well as their functional role. This knowledge is particularly relevant to disease prevention, since it helps define the epidemiological characteristics of new pathogen strains, and in some cases the extent of their virulence [1]. Current widespread concern over the potential threat of a human pandemic caused by mutated H5N1 avian influenza viruses highlights the medical, social, and economic value of tools that enable correct assessment of the potential for transmissibility of avian flu viruses amongst human hosts [2].

The influenza A virus is in equilibrium with its natural hosts, aquatic wildfowl, amongst which widespread transmission occurs, often without causing serious disease [3]. This virus has limited zoonotic potential: only four influenza subtypes have been known to circulate amongst humans, while at least 100 subtypes have been observed in birds. Domestic poultry and some mammals, particularly swine, are also hosts to a limited number of influenza A subtypes. However, occasional transmissions of influenza A to humans can have a tremendous impact. The Spanish flu pandemic of 1918/19 claimed over 40 million lives, and was almost certainly caused by adaptation of an avian H1N1 strain to humans [4]. Although the circulating H5N1 subtype has negligible potential for human-to-human transmission, there is a concern that it might acquire the necessary mutations for this capability.

Studies of the determinants of influenza host range and virulence have indicated that no single molecular factor can be pinpointed [5]. A multiplicity of mutations, distributed across several viral proteins, appears to be involved, making the experimental determination of the critical factors a complex task. A computational method is described in this paper, that compares arbitrarily large multiple sequence alignments of viral proteins and measures *mutual information *between the alignments at each amino acid site, leading to the identification of specific mutation patterns which characterize sets of sequences. *Characteristic variant patterns *of adaptation to human hosts can thus be identified by comparing human-to-human transmissible influenza strains to avian strains. *Sequence signatures*, which summarize the isolate-specific adaptation characteristics, can be extracted from these patterns. When ordered along a timeline, sequence signatures show the likely evolution of human-to-human characteristic mutations.

This paper describes the mutual information analysis method, and demonstrates its utility through an analysis of the influenza RNA polymerase protein PB2. This protein is a component of the ribonucleoprotein (RNP) complex, which is transported between nucleus and cytosol during viral infection and is therefore likely to require host adaptation. Indeed, specific mutations of the PB2 protein are known to participate in human-to-human transmission adaptation [6], and several studies have reported amino acid sites thought to be involved in this adaptation [7,8]. We used the results of these studies to show that mutual information analysis is highly effective for identifying systematic differences between sets of sequences. This method can be extended to the study of other pathogens, and of properties other than the host range.

Several methods for identifying the molecular determinants of pathogenic traits have been proposed [6-12]. *In vivo *and *in vitro *experiments are costly and time-consuming, and their scope is usually limited to the study of single mutations, rather than systematic screening. For example, Subbarao *et al*. [6] sequenced several influenza mutants of varying replication capabilities, which were derived from a single-gene reassortant virus and implicated a key role of a single mutation of the PB2 protein (E627K) in replication of influenza in humans.

*Ad hoc *computational methods involve production of multiple sequence alignments followed by visual inspection. Buckler-White *et al*. [9] identified 10 sites with distinctive human variants in influenza M1 and M2 genes by inspecting a handful of sequences. In a similar study, Naffakh *et al. *[7] identified seven sites in the PB2 gene from human variants from 34 aligned sequences. These studies lack statistical significance, which is a fundamental limitation of visual inspections: as the number of sequences increases, characteristic patterns become harder to discern. To alleviate this problem, researchers can split larger alignments into subgroups, using phylogenetic guide trees which cluster related sequences, making patterns more clearly visible (*e.g. *[10]). This approach was applied by Obenauer *et al*. [11] to define avian influenza *proteotypes *from alignments of up to 300 sequences, using a visual inspection method which is effective for sequence clustering, but cannot easily identify the residue mutation patterns that characterize each cluster. Visual inspection methods have additional drawbacks: they lack objective measures for assessing mutations, and they do not scale well-large-scale alignments make detailed inspections impractical. Both problems become particularly acute in sets characterized by multiple distinct mutations at the same site. The study of multiple distinct mutations can be addressed by formal methods, such as statistical diversity analysis based on information theory. Korber *et al*. [12] demonstrated the benefits of numerical variability measures by comparing the *information entropy *of separate alignments of HIV protein sequences, sampled from blood and brain tissues. They identified sites which were highly conserved (lower entropy) in the brain but not in blood, suggesting that the virus had forgone mutations to adapt to brain tissues. This method relies on entropy differentials between the two groups, and only sites characterised by high diversity in blood isolates were selected. However, this method is not capable of identifying sites which are conserved in blood isolates, but acquire mutations as a result of tissue adaptation.

A list of distinctive human variants of influenza A virus was compiled by Chen *et al*. [8], who identified sites with low entropy in both avian and human alignments, but with different consensus amino acids. By analyzing 401 full viral genomes, their study showed that entropy computations are highly scalable. However, information entropy alone is of limited use for comparative analysis, since it measures variability in a single alignment, without considering which variants occur in the other set. Of 52 characteristic sites reported by this study, eight were in the PB2 protein; however, several sites were discarded, because they exhibited either multiple variants or high variability in the avian set.

## Data analysis approach

### Characteristic sites and variants

Residues located at functionally important positions in a protein exhibit high conservation, since their mutations affect basic protein function and are usually detrimental to the organism's fitness. Conservation analysis is often used as a tool for the identification of functional residues [13,14]. This principle can be applied to functional components that confer specific properties to a pathogen population. For example, a mutation required for viral replication in a specific host must be conserved in host-adapted strains, but not in other strains. Such functionally important components can be therefore be found by comparing a *characterized set *of sequences (sequences selected on the basis of a common property), against a *reference set *(the pool of sequences that do not possess this property). This comparison can identify one or more *characteristic sites*: sites that exhibit residue variants which are common in the characterized set, but rare in the reference set, and are therefore likely to participate in conferring the defining property of the characterized set. In the present study, human-transmissible sequences (characterized set) have been compared to avian sequences which are not transmissible to humans (reference set). The sites identified in this analysis are therefore candidate functional sites responsible for human-to-human transmission. Our working hypothesis is that the poor transmissibility of most avian strains to humans could be accounted by the infrequent occurrence of human characteristic residues in avian sequences, at these sites.

### Entropy and mutual information

Information theory [15], defines variability measures such as *information entropy*, which are finding many applications in bioinformatics [16]. The entropy *H*(*x*) of a discrete random event *x*, whose possible outcomes form the set *E *= {*e*_1_, *e*_2 _... *e*_*n*_}, is a measure of the outcome uncertainty, given by:

$$H(x)=-\sum_{e\in E}p_{e}(x)\log_{2}\left(p_{e}(x)\right)$$

where *p*_*e*_(*x*) is the probability that *e *∈ *E *is the outcome of *x*. In protein alignments, we measure *residue entropy *by identifying *x *with a site, and *E *with the set of amino acids that occur at that site. Entropy values vary both with the number of amino acids observed at the site, and with their relative frequency. *H*(*x*) = 0 at sites with 100% conserved residues, while variable sites have higher entropy, up to a maximum of *H*_*max*_(*x*) = 4.322 (log_2_20). Such extreme variability is unlikely in closely related sequence sets and, in practice, sites whose residue entropy exceeds 1.0 can be regarded as highly variable.

Entropy can be used to measure variability in multiple alignments and identify conserved residues or peptide variants [17]. Because of its statistical nature, it is suitable for analyzing arbitrarily large dataset, and can thus be applied to large-scale diversity studies, such as the identification of stable antigenic targets over extended periods of time [18], a key step in reverse vaccinology [19].

Entropy computations can be combined to determine relationships between pairs of variables [15]. When considering two discrete events *A *and *B*, one can measure the *mutual information *(MI) of the two events as follows:

*MI*(*A*, *B*) = *H*(*A*) + *H*(*B*) - *H*(*A*, *B*)

where *H*(*A*, *B*) is the *joint entropy *of the two variables, which is computed using Equation (1), replacing *E *with the set of all unique pair of values (*A*, *B*).

MI is interpreted as the reduction in the uncertainty of the outcome of B when the outcome of A is known, and thus a measure of the dependence between the two variables. It was shown [20] that MI is 0 for two fully independent variables, while the MI of two variables that are fully co-dependent is determined by their entropy:

0 ≤ *MI*(*A*, *B*) ≤ min{*H*(*A*), *H*(*B*)}

MI has been used in mapping of genes and clustering of genetic markers [21]. It has also been employed to identify pairs of co-evolving sites in proteins, which produce high MI values when individual and joint residue entropies are combined using equation (2) [22].

### Mutual information of characteristic sites

We have utilized MI to identify characteristic sites in sets of aligned viral sequences. We compare pairs of *homologous alignments *to measure the relationship between the amino acids residues observed at a site, and the alignment in which they are observed. In a pair of homologous alignments, every residue site n in one alignment aligns with the same site n in the other alignment. In practice, pairs of homologous alignments may be formed by extracting sets of aligned sequences from a master alignment, without further realignment. Thus, variables *A *and *B *in equation (2) are replaced with the observed residue *a*, and the label *S *of the set (alignment) within which the residue is observed. The MI at a site *x *is therefore computed by:

*MI*(*x*) = *H*_*a*_(*x*) + *H*_*S*_(*x*) - *H*_*S*,*a*_(*x*)

*H*_*a*_(*x*) is simply the entropy at site *x *for the merged alignment, computed using (1). *H*_*S*_(*x*) is derived from the number of sequences in each of the two sets (*n*_1 _and *n*_2_):

$$H_{S}(x)=-\frac{n_{1}}{N}\log_{2}\left(\frac{n_{1}}{N}\right)-\frac{n_{2}}{N}\log_{2}\left(\frac{n_{2}}{N}\right)$$

where *N *= *n*_1 _+ *n*_2_. Finally, *H*_*S*,*a*_(*x*) is given by:

$$H_{S,a}(x)=-\sum_{S}\sum_{a\in A}p(S,a)\log_{2}p(S,a)$$

where *p*(*S*, *a*) is the probability of any given combination of residue and set label (in other words, occurrences of the same amino acid in two different sequence alignments constitute distinct outcomes).

Characteristic sites present different residues in the two sets, highly conserved within each set. Therefore, there is a strong relationship between residues and set labels at these sites, resulting in high MI values. Conversely, sites with low MI (approaching 0) exhibit similar distributions of amino acid variants in the two sets and are not characteristics. Since there are exactly two sets, the upper bound of *H*_*S*_(*x*) is 1, the maximum entropy for a variable with two outcomes. From equation (3) we therefore infer that 0 ≤ *MI*(*x*) ≤ 1. However, since *H*_*S*_(*x*) = 1 only when both alignments are equal in size, the range of *I*(*x*) is reduced when one set is larger than the other.

### Identification of characteristic sites and variants

A high MI value is the primary requisite of a characteristic site. However, the selection process must take into account a variety of factors that can reduce the MI. In influenza adaptation, human characteristic variants are sometimes present in a minority of avian strains, which is expected if the mutations necessary for human adaptation originate in the avian pool. Furthermore, sporadic random mutations and episodes of infections from other hosts can be observed in both sets. Finally, avian characteristic variants are expected to be present in historical sequences, sampled before these variants stabilized. To select characteristic sites and variants, we identified four criteria that help distinguish characteristic sites from the background noise. The choice of threshold values for these criteria is largely dependent on the analysis task selection. The four criteria are:

• A characteristic site *s*_*c *_must have an MI value above *MI*_*min*_, the MI threshold below which no characteristic sites are deemed to be present.

• If a characteristic variant *v*_*c *_is present at site *s*_*c *_with probability *pc*(*v*_*c*_, *s*_*c*_) within the set it represents and *po*(*v*_*c*_, *s*_*c*_) in the other set, the ratio *r*(*v*_*c*_, *s*_*c*_) = *pc*(*v*_*c*_, *s*_*c*_)/*po*(*v*_*c*_, *s*_*c*_) must exceed a minimum frequency ratio *r*_*min *_if *po*(*v*_*c*_, *s*_*c*_) is non-zero. A high *r*_*min *_ensures that the variant is significantly more common in the set it represents.

• The probability *pc*(*v*_*c*_, *s*_*c*_) must exceed a minimum probability *pc*_*min*_. Raising this threshold prevents statistically insignificant mutations from being considered characteristic, even when they are more frequent in one set than in the other.

• At a characteristic site *s*_*c*_, the probability *pc'*(*S*, *s*_*c*_) of a set *S *containing variants characteristic of the other set must be lower that the maximum contamination probability *pc'*_*max*_(*S*). This threshold prevents a site from being classified as characteristic if there is significant cross-contamination of variants between the two sets. Depending on the analysis task, it is desirable to specify a different threshold for each set: for example, the tolerance for human variants present in avian sequences may be greater than the tolerance for avian variants in human sequences, to account for a more diverse pool of mutations in the avian virus population.

The selection process produces a *characteristic variant pattern*: a catalogue of characteristic sites, each possessing a list of the characteristic variants identified for each of the two sets. A characteristic variant pattern therefore presents in a concise form the systematic differences between a pair of aligned sequence sets, and can be used to derive a *sequence signature *for any homologous sequence. Sequence signatures comprise only the residues at characteristic sites, and thus provide a concise representation of any given isolate, useful for determining which characteristic mutations it possesses.

### Experimental considerations

When choosing a *MI*_*min *_threshold, it is desirable to apply the same threshold values to multiple different comparisons (for example, for all the influenza proteins). However, there are several statistical factors that influence MI values between alignments. These factors include: the presence of alignment gaps; the number of sequences in the comparison sets; and the size ratio of these sets.

Alignment gaps, introduced by alignment algorithms, need to be accounted for in MI computations. The gap symbol is an artifact of the alignment and therefore cannot be considered an additional amino acid. However, its presence affects entropy calculations: although gaps are indicative of high variability, they artificially lower the entropy of sites where they are numerous [23]. Although it is possible to estimate entropy at highly gapped sites, one has to question whether it is meaningful to seek characteristic variants at highly variables sites, where a large proportion of the sequences have no residue at all. A *maximum gap threshold *should therefore be chosen (for example, 50%), above which a position should be ignored for MI computation purposes.

**Figure 1:**
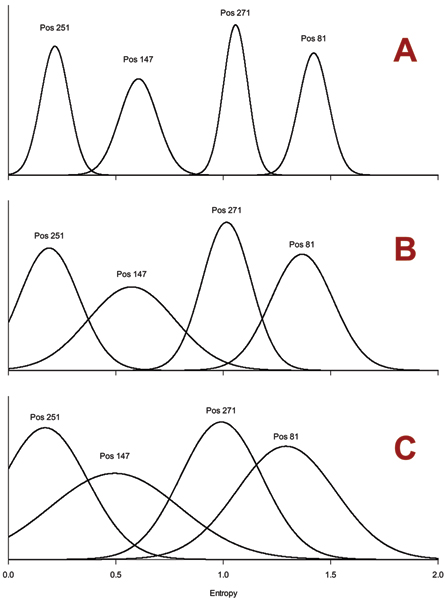
**Effect of set size on information entropy**. The probability density of entropy values at four sites of the Influenza A PB2 proteins is plotted for alignments of decreasing sequence count N (graph A: N = 250; graph B: N = 50; graph C: N = 20). For each graph, we constructed 200 random alignments of the required size from the PB2 master alignment. The entropy mean and standard deviation measured from these alignments were used to plot the normal probability distributions shown in this chart. The entropy values for different sites are well-separated in large sequence sets (plot A) while the likelihood of distinguishing medium-entropy sites from high- or low-entropy sites drops dramatically at low sequence counts (plot C). The sites were selected based on their equally-spaced entropy values.

The number of sequences in a given set affects the computed entropy values and hence MI values. Entropy is a statistical measure, and thus most accurate as sequence count approaches infinity. Previous studies [24] indicate that smaller set sizes introduce an error which is inversely proportional to the sequence count. Our own experiments show that, when averaging over several random subsets, this relationship holds for sets as small as 20 sequences. However, for very small sequence counts the dramatic increase in sampling error makes entropy-based comparisons unreliable (Figure 1). In the present study, we only used sets of 50 sequences or more.

**Figure 2:**
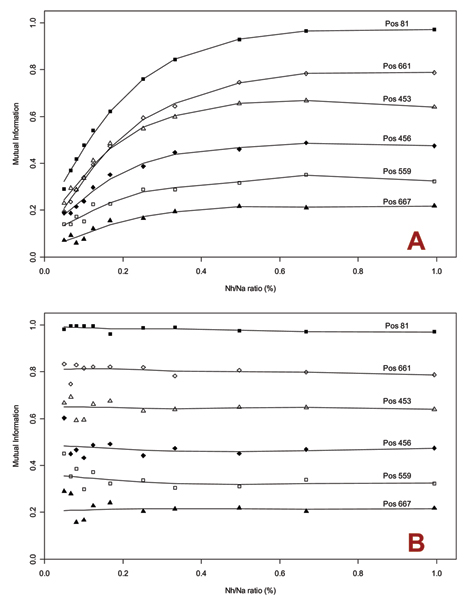
**Effect of set size bias on mutual information**. In both graphs, the y-axis represents the measured mutual information (MI) between two sets of influenza A PB2 protein sequences, comprising human and avian sequences respectively. The x-axis represents the size ratio Nh/Na, where Nh and Na are the sequence count in the human and avian sets respectively. A) Changes in MI at selected alignment sites as Nh is varied (Na = 719). MI values fall rapidly as the ratio decreases, especially at sites with high MI. B) Each data point is computed by averaging the MI obtained by comparing the human set with 200 random subsampled sets of avian sequences with the same sequence count. The estimated MI values remain stable up to a size ratio of approximately 1:10. At very low ratios, increased sampling errors due to small set size tend to lower the reliability of the estimate.

As previously discussed, any size disparity between the two sequence sets being compared reduces the range of MI values. Figure 2-A shows that, as one set becomes several times larger than the other, MI values decrease at all sites. This relative size bias is problematic if characteristic site selection relies on absolute thresholds. When size disparity exists, we therefore correct for this bias using a sampling method, which compares the smaller of the two sets to multiple subsets of the larger set and evaluates the mean MI. Each subset is randomly selected and equal in size to the smaller set of aligned sequences. Figure 2-B shows the effect of this correction: MI values remain stable even as set size ratio exceeds 1:10, especially at sites with high MI. Small sequence counts, however, affect the estimate reliability at very low ratios. These measurements indicate that the sampling correction gives reliable MI results with size ratios up to 1:10.

### The role of metadata

Multiple alignments used in comparative studies are constructed from sequences selected according to common characteristics, for example human-to-human or avian-to-avian transmission of infection. For small numbers of sets with well-defined selection criteria, alignments can be produced from separate queries to public databases such as Genbank. However, matching the positions of separately-produced alignments is often problematic due to the presence of gaps, which are introduced by the alignment algorithm and whose position may vary in different alignments. In practice, comparative studies often need to test multiple hypotheses that require diverse selection criteria (for example, restricting selections to specific time periods, or geographical areas), demanding considerable additional effort in alignment construction. To address this problem, we constructed an annotated dataset in which sequences are accompanied by descriptive metadata, including strain name, subtype, host, year and country of isolation, protein name. We produced master alignments for each protein, so that subset alignments can be subsequently extracted through metadata queries, without further realignment. This method allows rapid comparisons of sequence subsets using arbitrary selection criteria.

Quality-controlled metadata is difficult to obtain from public sequence databases, since the annotations of a large proportion of sequence records are inconsistent, incomplete, or even erroneous [25]. Several approaches have been proposed to address these problems [26,27], many of which require significant computing infrastructure or programming knowledge. This study made use of the Aggregator of Biological Knowledge (ABK) [28], a desktop tool that employs user-defined *structural rules *to extract values from multiple annotation fields and from multiple sequence records, and subsequently reconcile conflicts in the extracted metadata. By automating the metadata extraction process, this tool enables the rapid construction of very large sequence datasets. More than 85,000 influenza records were processed and annotated with relevant high-quality metadata for the current study. The use of structural rules enabled us to complete this task with only modest requirements for manual curation effort.

### Metadata-enabled analysis: the AVANA tool

**Figure 3:**
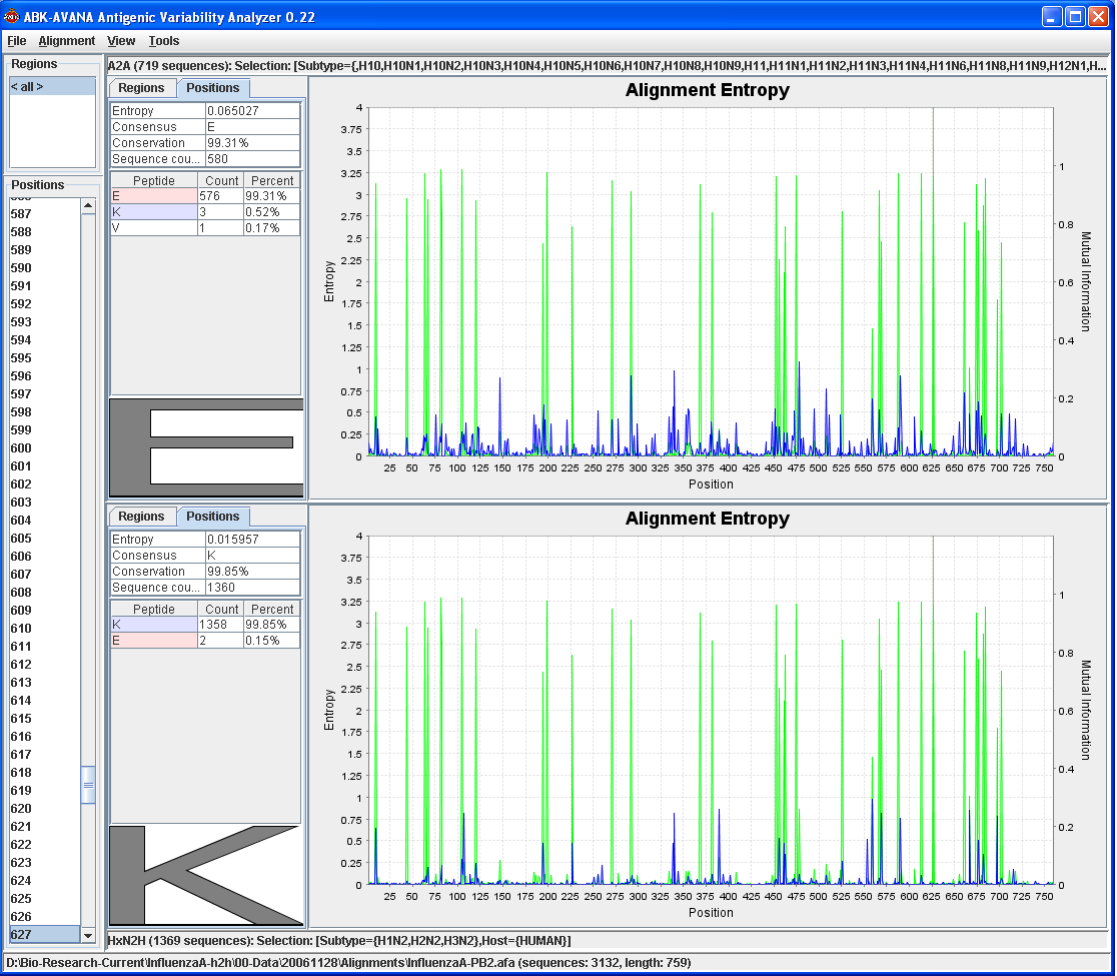
**Screenshot of the Antigenic Diversity Analyzer (AVANA)**. This screenshot shows the AVANA tools used in a comparison of the A2A (top) and HxN2 (bottom) subsets. The horizontal axis corresponds to the positions along the alignment, while the vertical axes represent the entropy of each subset (blue), and mutual information (green) between the two subsets. Characteristic sites are identifiable by the presence of MI peaks. On the left-hand site, AVANA displays the residue statistics at the currently selected position: the E627K characteristic mutation is shown in this example.

The Antigenic Variability Analyzer (AVANA) tool, which supports a variety of entropy-based analyses of multiple sequence alignments, is the software engine used to support this study. This tool calculates and plots entropy profiles for multiple sequence alignments, allowing users to inspect variants and their frequencies at each position. AVANA can analyze the variability of peptides of any lengths, which makes it suitable for studying antigenic characteristics of pathogens [18]. For maximum flexibility, the tool is able to include arbitrary metadata fields (annotations), and select subsets of the master alignment using metadata values. Since metadata is loaded separately from the master alignment, the latter can be produced using the multiple sequence alignment tool of choice. MI computation, size bias adjustments and characteristic site identification are built into the AVANA toolset, which automates the identification of characteristic variant patterns and produces sequence signatures. Figure 3 shows a screenshot of the AVANA tools, which can be deployed on any Java-enabled operating systems, and is freely available upon request to the authors.

## Results and discussion

### Catalogue of characteristic sites

**Figure 4:**
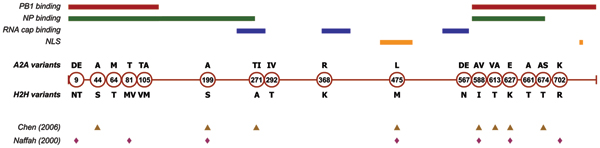
**Characteristic sites for human-to-human transmission (H2H) identified in the PB2 protein of the influenza A virus**. The sites, whose position is indicated in the circles, are arranged along the length of the protein, with the avian (A2A) variants and the H2H variants indicated above and below the circles respectively. Where multiple variants are present at a site, they are shown in decreasing order of frequency. The coloured lines in the upper part of the figure show the extent of identified PB2 functional domains: the binding regions of PB2 to the PB1 and NP proteins [33] are shown in red and green respectively; the RNA cap binding regions [34, 35] in blue; and the nuclear localization signals (NLS) [32] in orange. Except for site 292, all characteristic sites identified are within one, or two functional domains. The lower part of the figure shows characteristic sites previously identified in other studies [8, 7].

A total of 17 characteristic sites were identified in this study (Figure 4). These 17 characteristic sites are present in both subtypes currently circulating amongst humans (H1N1 and H3N2). All H2H characteristic variants exhibit extremely high conservation in humans (>99% except at position 613 where conservation is 96.8%), which is indicative of a possible role in the adaptation mechanisms of influenza A virus to human hosts. High conservation at characteristic sites is also typical in avian sequences (>95%), but not as uniformly, as some H2H characteristic variants appear with low frequency in the avian population (full details are shown in Table 1). Most discovered sites showed very little presence of human variants in avian sequences, with one notable exception: at site 702 the human variant (arginine) was present in 10% of avian sequences. This variant appears to be common in H9N2 avian viruses, which are known to infect humans [29]. However, the same variant was not present in the H9N2 strain A/quail/HongKong/G1/97, thought to have originated the PB2 gene of the H5N1 strains that claimed human lives in Hong Kong in 1997 [30].

**Table 1:** Full catalogue of characteristic sites for human-to-human transmission of influenza A identified for the PB2 protein. For each site, we show the position in the alignment; the characteristic variants of the avian-to-avian (A2A) and human-to-human (H2H) transmissible sequences; the characteristic variants found in human H1N1 and HxN2 subtypes; the year when the human characteristic variant was first isolated; the conservation of the characteristic variants in avian and human sequences; and finally the percentage of human sequences that exhibit avian variant. Sites where human characteristic variants were present in 1918 are highlighted in bold type.

**Position**	**Char. Variants**		**1st Human isolate**	**Conservation**		**X-presence of A2A**
				
	**A2A**	**H2H**		**A2A**	**H2H**	
9	DE	NT	1933	98.57%	99.33%	0.49%
44	A	S	1940	96.82%	99.27%	0.61%
64	M	T	1933	97.29%	99.58%	0.30%
81	T	MV	1933	97.93%	99.27%	0.30%
105	TA	VM	1933	98.41%	99.45%	0.36%
**199**	**A**	**S**	**1918**	**99.47%**	**99.76%**	**0.24%**
271	TI	A	1940	98.59%	99.51%	0.37%
292	IV	T	1940	95.54%	99.15%	0.67%
368	R	K	1940	98.12%	99.33%	0.67%
**475**	**L**	**M**	**1918**	**99.66%**	**99.76%**	**0.24%**
**567**	**DE**	**N**	**1918**	**98.28%**	**99.39%**	**0.55%**
588	AV	I	1940	98.45%	99.63%	0.31%
613	VA	T	1940	98.28%	96.82%	0.61%
**627**	**E**	**K**	**1918**	**99.31%**	**99.76%**	**0.12%**
661	A	T	1933	86.72%	99.39%	0.43%
674	AS	T	1933	95.69%	99.63%	0.18%
**702**	**K**	**R**	**1918**	**89.70%**	**99.39%**	**0.49%**

Previous studies have reported several of these sites as possible determinants of host-range specificity. The E627K mutation affecting influenza replication in humans [6], was also associated with the high virulence of human H5N1 infections [31]. Seven characteristic sites in PB2 were identified by Naffakh *et al. *[7], while Chen *et al*. [8] reported eight sites. Combined, all these studies identified 11 sites (Figure 4), all of which were also found in the present study. All except one of the 17 characteristic sites reported herein are found in experimentally determined functional domains of the PB2 protein: signals controlling translocation to cell nuclei [32], binding sites for proteins in the polymerase complex [33] or RNA cap binding sites [34,35]. This suggests that these mutations could play a role in the adaptation of critical viral functions to human hosts.

The high number of characteristic mutations, and their location in areas of contact with other proteins, suggest that H2H adaptation relies on complex interactions, and that the contribution of individual mutations may difficult to quantify and demonstrate experimentally. In the absence of experimental evidence, one cannot discount the possibility that some of the mutations identified have "hitch-hiked" alongside functionally important mutations. It must be noted, however, that all characteristic sites identified have remarkably stable in humans over a period of nearly 70 years, which suggests that they are important components of the adaptation, or that they play a supports role to functionally important mutations.

In addition to the 17 characteristic sites common to both H2H groups, we identified two characteristic sites unique to the H1N1H set, and nine unique to the HxN2H set (Table 2). Conservation is somewhat lower at these sites, possibly because they emerged at a later date.

**Table 2:** Catalogue of subtype-specific characteristic sites for human-to-human transmission of influenza A identified for the PB2 protein. The upper table shows characteristic sites exhibited only by H1N1 human viruses, while the lower table lists characteristic sites common to H2N2, H1N2 and H3N2. For each site, we show the same values as in Table 1, except that the human variants are subtype-specific.

**Position**	**Char. Variants**		**1st Human isolate**	**Conservation**		**X-presence of A2A**
				
	**A2A**	**H1N1**		**A2A**	**H1N1**	
**114**	**V**	**I**	**1918**	**100.00%**	**98.57%**	**1.43%**
491	T	A	1933	98.94%	97.48%	2.52%

**Position**	**Char. Variants**		**1st Human isolate**	**Conservation**		**X-presence of A2A**
				
	**A2A**	**HxN2**		**A2A**	**HxN2**	

67	I	V	1964	99.68%	97.01%	2.99%
82	N	S	1957	94.27%	97.15%	0.95%
120	E	D	1970	99.52%	96.20%	3.73%
382	I	V	1961	96.77%	98.53%	1.47%
453	PS	H	1940	98.29%	99.49%	0.29%
526	K	R	1972	99.65%	95.66%	4.34%
676	TA	I	1965	96.21%	91.47%	4.93%
682	G	S	1972	100.00%	94.56%	4.19%
684	A	S	1957	96.90%	99.63%	0.22%

### Evolutionary timelines

**Figure 5:**
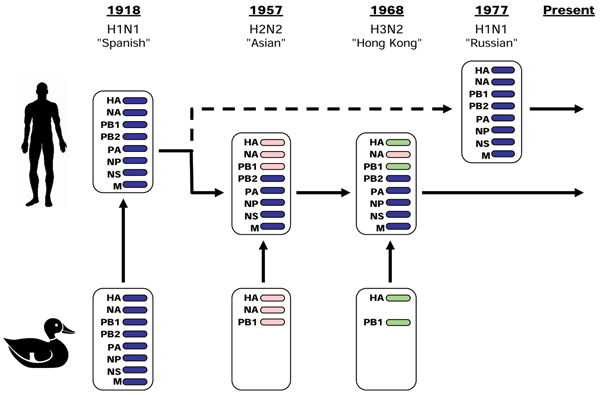
**Evolution and reassortment of human Influenza A viruses**. This figure (adapted from [3]) shows how human-transmissible Influenza A subtypes were acquired from the avian pool during 20^th ^Century pandemics. A full complement of eight gene segments of avian origins originated the 1918 Spanish flu, while the following two pandemics followed the acquisition of a smaller number of avian genes through recombination. In 1957, the H2N2 Asian flu replaced the HA, NA and PB1 segments, while the H3N2 Hong Kong pandemic of 1968 replaced the HA and PB1 segments only. In each of these pandemics, the new subtype fully replaced the previously circulating subtype. The minor Russian pandemic of 1977 was caused by the reintroduction of a H1N1 strain almost identical to that circulating prior to 1957, leading to the widely-held view that it was caused by the release of 20-year old frozen viruses. The H1N1 strain has not supplanted H3N2, and the two lineages co-circulate in the human population to the present day; the recently emerged H1N2 subtype has arisen from their reassortment. All currently circulating PB2 proteins are therefore thought to have descended from the Spanish flu strain, although the PB2 protein associated with HxN2 has diverged significantly from that of the H1N1 lineage.

**Figure 6:**
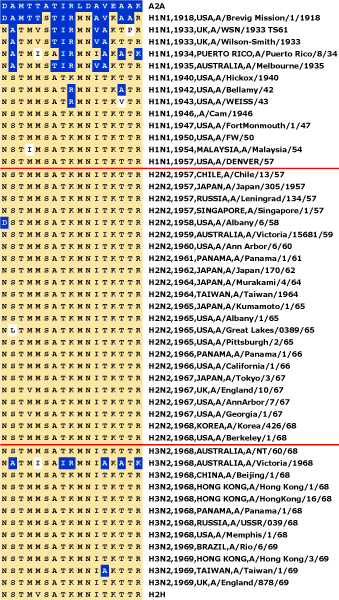
**Timeline of adaptation to human-to-human transmission for the influenza A PB2 protein**. Using the H2H characteristic variant pattern (see Fig. 4), we produced signatures for each available human sequence isolated before 1970, and arranged them in chronological order. The signature columns show the residues observed at each of the characteristic sites, in the order given in Figure 4. Each signature is annotated with subtype, year and country of isolation, and isolate name. The first and the last pattern of the alignment are the consensus signatures for avian and human-to-human transmissible sequences respectively. Avian characteristic variants are shown on a dark blue background, human characteristic variants on a yellow background, and all other variants are on white. Red horizontal lines indicate the start of the 1957 and 1968 pandemics, which introduced the H2N2 and H3N2 subtypes respectively. The GenPept accession numbers for all sequences used are listed in Table S1 in Additional file 1.

To reconstruct the emergence of characteristic mutations, we used the characteristic sites catalogue to produce signatures for all human PB2 sequences isolated between 1918 and 1970, a the period spanning over the three major 20^th ^Century pandemics (see Figure 5). The signatures were arranged chronologically to form a timeline, as shown in Figure 6. Coloured backgrounds, distinguishing avian and human characteristic variants, permit an intuitive visualization of the emergence of adaptation to humans. Although the signature of the virus strain responsible for the 1918 Spanish flu was clearly avian, it contained five H2H variants (including site 627). By comparison, the signatures of all 1174 avian (A2A) sequences revealed only one sequence (H9N2) containing as many as three H2H variants, with 77% of sequences containing no H2H variants.

Fifteen years later, the signature of the H1N1 circulating in the human population had acquired a predominantly human signature, and viruses isolated in 1940 had signatures identical to circulating strains today. In summary, the characteristic variant pattern of human-to-human transmission evolved fully with remarkable speed (20 years), and has shown great stability over the following 65 years. The timeline shows that the two pandemics of 1957 and 1968 (which introduced the H2N2 and H3N2 subtypes respectively) had no effect on the continuity of the H2H signature for PB2, although both pandemics were probably zoonotic in origin. This supports the widely accepted notion that these pandemics involved reassortment of avian strains with human-adapted strains, in which the PB2 protein which originally evolved from the 1918 H1N1 strain was retained [36]. At first sight, a common origin of PB2 in all three subtypes seems to contradict the different variant patterns of H1N1H and HxN2H. However, H1N1 reappeared unchanged, twenty years after its elimination by the 1957 pandemic, possibly due to accidental release from a laboratory [3,37]. The PB2 protein of this strain, effectively the ancestor of the H3N2 PB2, was thus re-introduced to form a separate lineage.

**Figure 7:**
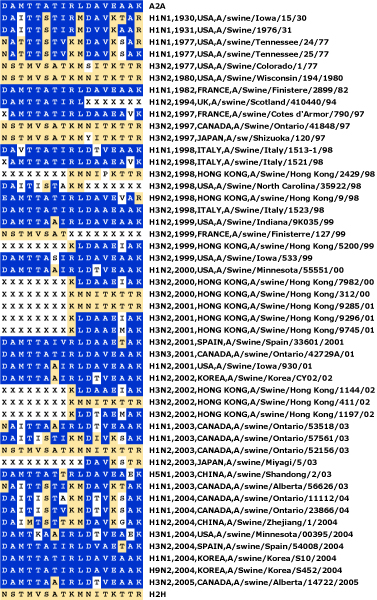
**Signatures of swine-isolated influenza A PB2 proteins against the H2H characteristic variant pattern**. The H2H characteristic variant pattern is the same as that used in Figure 6. The symbol 'X' at a characteristic site indicates that the residue is unknown, due to an incompletely sequenced protein. Some patterns, whose signatures are represented by other retained sequences, were removed from the alignment to make the figure more compact. The GenPept accession numbers for all sequences used are listed in Table S2 in Additional file 1.

The timelines obtained can be useful for detecting episodes of zoonotic infections, since the signature of avian and swine sequences are noticeably different from human signatures. The signatures of recent human isolates reveal that A/Hong Kong/1774/99 and A/Ontario/RV1273/2005 strains possessed PB2 proteins with avian characteristics, a sign of avian-to-human transmission, perhaps via a swine intermediary [38]. In addition, our results show that both human and avian characteristic variants circulate amongst pigs (Figure 7), making this group of sequences singularly difficult to characterize.

### Characterization of H5N1

**Figure 8:**
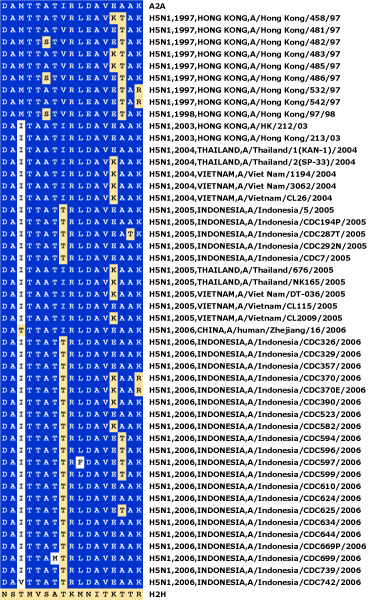
**Signatures of human-isolated H5N1 influenza A PB2 proteins against the H2H characteristic variant pattern**. The H2H characteristic variant pattern is the same as that used in Figure 6. Some patterns, whose signatures are represented by other retained sequences, were removed from the alignment to make the figure more compact. The GenPept accession numbers for all sequences used are listed in Table S3 in Additional file 1.

Figure 8 shows a timeline of H2H signatures derived from human H5N1 isolates. All signatures are clearly avian although, unlike the majority of avian strains, most contain at least one human variant. The presence of human variants may help explain the multiple occurrences of avian-to-human infections. However, the timeline suggests that H5N1 strains are not accumulating mutations that will increase their potential for stable adaptation to humans: even the E627K mutation, present in some sequences, is not conserved.

This finding is in agreement with the extreme rarity of human-to-human transmission of avian influenza viruses. It is also consistent with the observation that the 1957 and 1968 pandemic viruses needed to acquire most of their internal genes from reassortments with human-adapted strains. Even if H5N1 became capable of a similar reassortment with human strains, its potential for pathogenicity could be affected in unpredictable ways. Since studies have failed to identify a single molecular component responsible for H5N1 pathogenicity, it appears that the pathogenicity is systemically determined, and that internal proteins may be involved.

### Metadata availability issues

The method described in this study is generic, and applicable to any analysis of systematic differences between sets of homologous sequences, selected on the basis of a particular metadata field. For example, the emergence of new characteristics can be studied by comparing sequence sets representing different periods of time. The integration of metadata capabilities in the analysis tools enables the rapid analysis of multiple sequence subsets, with dramatically reduced data preparation effort. Sequence metadata is underutilized in current bioinformatic data mining approaches. We have shown that analysis tools can be greatly enhanced by this additional knowledge. However, the quality of results depends on the quality of the underlying metadata. Collecting annotations is currently a significant obstacle for large-scale analysis, largely due to the uneven quality of annotations in large public databases. Large-scale efforts, such as the NIAID project [39], and specialized databases, such as NCBI Influenza Virus Resource , are improving the consistency of influenza A annotations. Text mining techniques can support metadata gathering by analyzing publications associated with sequence records. The availability of intuitive and reusable text mining tools is increasingly useful for extracting annotations for specific purposes [40]. In the long term, however, problems of data duplication and inconsistencies in large-scale public databases are likely to persist. There is a need to complement public molecular databases with well-curated high-quality annotations. Knowledge management approaches, such as Semantic Web technologies [41], are likely to prove helpful in this area.

## Conclusion

This paper presents a novel approach to the identification of characteristic variant patterns, based on the comparison of pairs of sequence alignments. We have shown that the method has important practical applications, including the identification of host range determinant mutations in Influenza A viruses.

The higher number of PB2 characteristic sites identified in the present study with respect to previous studies show that mutual information analysis is more powerful than earlier methods applied for this purpose. This is largely due to the enhanced comparative power and high scalability of the statistical measures employed. The positions of the identified characteristic sites indicate their potential functional significance: 16 of 17 characteristic sites are located in the well-defined functional domains of PB2 protein. Characteristic variant patterns are a useful tool for interpreting historical data. Sequence signatures derived from characteristic variant patterns provide for a concise and understandable representation of individual sequences and evolving strains, while the timelines assembled from signatures of historical sequences are a helpful tool for understanding the emergence of specific characteristics. This method is generic and can be applied to any studies where it is desirable to perform molecular characterization of sequence groups from large-scale analysis, particularly for organisms with a high level genetic variability.

### Future work

A further possible application of the MI approach is in genotyping, which typically relies on the interpretation of phylogenetic trees, usually involving a strong subjective component. Mutation patterns often characterize genotypes [11], and mutual information analysis can provide an objective measure of clustering goodness, while characteristic variant patterns and sequence signatures can be used to classify sequences.

## Methods

### Data collection and preparation

We conducted a study on a set of influenza A sequences annotated with the following metadata properties: isolate name, country and year of isolation, host organism, subtype, and protein name. To include as much historical information as possible, the working dataset was derived from all available sequences (as of September 2006) from the NCBI GenBank and GenPept databases [42], including entries mirrored from UniProt [43]. Data collection and cleaning was performed using the ABK tool, which processed a total of 85,873 records retrieved using taxonomy-based queries. ABK merged complementary annotations from DNA and protein records, checked them for consistency and removed duplicate entries. The resulting annotations were verified manually by two independent curators, producing a set of 40,169 amino acid sequences, grouped by protein. Both full-length and fragment sequences were used, since entropy measurements do not require that all sequences contribute to entropy at all sites.

The analysis described herein focused on a master set of 3,132 annotated PB2 polymerase protein sequences. The master alignment was carried out using MUSCLE 3.6 [44]. AVANA was subsequently used to extracts alignments of specific subsets of the collected sequences, based on annotation values. Since the subsets were extracted from the master alignment without realignment, a direct comparison of residue statistics could be made at each site.

### Identification of distinctive sites and reconstruction of evolutionary timeline

A catalogue of characteristic sites for human-to-human transmission was prepared using alignments of three subsets of protein sequences:

• A2A: the subset of all avian sequences of all subtypes, excluding those known to be transmissible amongst humans (H1N1, H2N2, H1N2 and H3N2). The H5N1 subtype, which is known to infect humans, was also excluded (719 sequences)

• H1N1H: the subset of all human sequences of H1N1 subtype (281 sequences)

• HxN2H: the subset of all human sequences of H2N2, H1N2 and H3N2 subtypes (1369 sequences)

The H2N2, H1N2 and H3N2 subgroups are grouped into a single HxN2H subset, because their PB2 proteins are known to share a common lineage [3]. Human H1N1 sequences are grouped separately, since they constitute a separate co-circulating lineage [3], as evidenced by phylogenetic analysis [45].

The AVANA tool analysis produced two catalogues of characteristic sites, from comparisons of A2A to H1N1H, and A2A to HxN2H. In both case, the same set of analysis parameters was used, determined as follows.

• The threshold *MI*_*min *_= 0.4 was determined by an analysis medium-MI sites in all internal proteins of influenza, which indicated that avian and human sequences converge to the same consensus amino acids as MI falls below 0.4 (data not shown).

• To identify characteristic variants, the threshold *r*_*min *_= 4 was chosen (*i.e. *characteristic variants must be four times more frequent in the set they characterize than in the other set). To determine this value, we analyzed the probability ratio *r*(*v*, *s*) for all variants at each position in the alignment (discarding variants with >99% conservation, or probabilities below 1%). For PB2, the standard deviation of *log*_10_*r*(*v*, *s*) was 0.52, corresponding to a ratio of 3.29 (the log transformation was applied so that ratios could be compared on a linear scale). An identical analysis of an alignment of NS1 protein sequences produced a consistent ratio of 3.26, although NS1 is the most variable internal influenza A protein. A slightly more conservative threshold ratio of 4 was chosen for our analysis. Post-analysis verification confirmed that no H2H characteristic variant presented ratios lower than 9.65, while the highest ratio among H2H non-characteristic variants was 1.45.

• To classify characteristic variants, we chose a threshold value *pc*_*min *_= 0.02 (*i.e. *characteristic variants must occur in at least 2% of the sequences), which translates to a minimum support of approximately 30 sequences for H2H characteristic variants. Post-analysis verification showed that the lowest support for characteristic variants was 65 sequences (residue M at site 105), while support for non-characteristic human variants at characteristic sites never exceeded 11 sequences (with the exception of residue I at site 613, which was supported by 41 sequences, but was discarded because of insufficient frequency ratio *r *= 1.45). These results indicate that no important characteristic variant was omitted by our choice of threshold.

Rather than set arbitrary limits for *pc'*_*max *_(the maximum contamination in each of the two sets), we manually inspected sites that presented contamination higher than 2%. All characteristic sites identified in both human sets had less than 5% contamination from avian variants. Above this value, the sites identified were discarded, as they were only characteristic of one human lineage, usually because they emerged long after the lineage became established (for example, variants developed by H1N1 after its reintroduction in 1977).

The H1N1H and HxN2H catalogues were combined, selecting only positions contained in both catalogues to produce a characteristic variant pattern of human-to-human (H2H) transmission. To investigate the evolutionary timeline for the emergence of H2H characteristic variants, a subset alignment (named HPre1970) was constructed, comprising all human sequences sampled before 1970. This alignment was processed by the AVANA tool, which extracted the signatures of each sequence against the H2H characteristic variant pattern, and arranged these signatures in chronological order. The same analysis was carried out for other sequence subsets, notably the set of all H5N1 sequences isolated in humans (H5N1H).

## Competing interests

The authors declare that they have no competing interests.

## Authors' contributions

OM developed the analysis methodology and applied it to the influenza dataset. OM and ATH jointly collected and annotated the sequence data. TWT and VB provided advice and contributed to the study design and interpretation of results. JTA coordinated this study and contributed to the interpretation and discussion of results. All authors critically reviewed and approved the final manuscript.

## Supplementary Material

Additional file 1 Tables S1 to S3 – GenPept accession numbers for all sequences used in Figures 6, 7 and 8. Click here for file
