# Supplementary material for: Identification of human-to-human transmissibility factors in PB2 proteins of influenza A by large-scale mutual information analysis
Source: BMC Bioinformatics. 2008 Feb 13;9(Suppl 1):S18. doi: 10.1186/1471-2105-9-S1-S18 (PMC2259419; doi:10.1186/1471-2105-9-S1-S18)
Supplement: Additional file 1 — Tables S1 to S3 – GenPept accession numbers for all sequences used in Figures 6, 7 and 8. [file 1471-2105-9-S1-S18-S1.pdf]

Olivo Miotto, AT Heiny, Tin Wee Tan, J Thomas August, Vladimir Brusic

Identification of human-to-human transmissibility factors in PB2 proteins of influenza A by large-scale mutual information analysis

Additional Materials

Contents

Table S1. List of GenPept accession numbers for the sequences used to produce the timeline in Figure 6 (Human sequences).

Table S2. List of GenPept accession numbers for the sequences used to produce the timeline in Figure 7 (Swine sequences).

Table S3. List of GenPept accession numbers for the sequences used to produce the timeline in Figure 8 (Human H5N1 sequences).

Table S1. List of GenPept accession numbers for the sequences used to produce the timeline in Figure 6 (Human sequences). The sequences are listed in the order in which they appear in Figure 6.

ABA55038.1
ABF47965.1
ABD77806.1
ABD77685.1
ABD62791.1
ABI20836.1
ABD62852.1
ABD79111.1
ABD79122.1
ABD77817.1
ABD61745.1
ABD60976.1
ABD15269.1
AAO46250.1
ABI84969.1
AAA19213.2
AAA43127.1
AAO46252.1
AAO46253.1
AAO46254.1
AAO46255.1
AAO46256.1
AAO46257.1
ABF21233.1
AAO46259.1
AAO46258.1
AAA43595.1
AAO46260.1
AAO46261.1
AAO46262.1
AAO46266.1
AAO46264.1
AAO46263.1
AAO46265.1
YP_308855.1
AAO46267.1
AAA43613.1
ABI92290.1
ABC84399.1
AAK51712.1
AAO46493.1
AAO46494.1
AAO46495.1
ABB54524.1
AAO46496.1
ABB80044.1
AAO46497.1
AAO46498.1

Table S2. List of GenPept accession numbers for the sequences used to produce the timeline in Figure 7 (Swine sequences). The sequences are listed in the sequences in which they appear in Figure 7.

AAA43126.1
AAA43649.1
AAA43125.1
ABD95721.1
ABD61561.1
ABD61259.1
CAC36998.1
CAC84738.1
CAC84739.1
AAG01757.1
AAO15321.1
CAC84745.1
CAC84743.1
AAL30473.1
AAD51247.1
AAL14086.1
CAC37000.1
AAF76002.1
CAC84741.1
AAR12289.1
AAG01775.1
AAL87932.1
AAR12291.1
AAR12280.1
AAR12281.1
AAR12292.1
AAR12293.1
ABD62842.1
AAU25862.1
AAL87933.1
AAN46834.1
AAR12294.1
AAR12285.1
AAR12295.1
ABB86900.1
ABB86870.1
ABB86910.1
BAF03572.1
AAU05323.1
ABB86880.1
ABB86940.1
ABB86930.1
AAZ79398.1
ABA46960.1
ABE27174.1
AAV67991.1
AAV68028.1
ABF18003.1

Table S3. List of GenPept accession numbers for the sequences used to produce the timeline in Figure 8 (Human H5N1 sequences). The sequences are listed in the sequences in which they appear in Figure 8.

CAB95862.1
AAK49373.1
AAF74311.1
AAF74312.1
AAF74313.1
AAK49376.1
AAK49377.1
AAK49379.1
AAK49380.1
AAT39029.1
BAE07198.1
AAV35117.1
AAV32653.1
AAT73549.1
AAT73552.1
ABF01745.1
ABI35999.1
ABI36213.1
ABI36223.1
ABI36227.1
ABI36008.1
ABC72647.1
ABD16291.1
AAZ72955.1
ABF01749.1
ABF01751.1
ABG23659.1
ABI36234.1
ABI36248.1
ABI36254.1
ABI36259.1
ABI36263.1
ABI36274.1
ABI36194.1
ABI36305.1
ABI36140.1
ABI36162.1
ABI36173.1
ABI36306.1
ABI36311.1
ABI36344.1
ABI36366.1
ABI36377.1
ABI36482.1
ABI36443.1
ABI36454.1
ABI49392.1
ABI49403.1
